# Using Governance to Elevate Voluntary and Community Sector Representation

**DOI:** 10.5334/ijic.9121

**Published:** 2025-07-25

**Authors:** Andrea Bentz, Elizabeth Tanguay, Dania Versailles, Sylvie Lefebvre

**Affiliations:** 1 University of Ottawa, Ontario, Canada; 2 Ottawa, Ontario, Canada; 3 Archipel Ontario Health Team, Ottawa, Ontario, Canada

**Keywords:** integrated care, governance, intersectoral, shared leadership

## Abstract

Archipel Ontario Health Team (OHT) developed as part of the provincial call to organize health and social service providers into integrated care delivery systems. With nearly 70 multi-disciplinary and multi-sectoral partners, unequal power dynamics must be considered when creating governance structures. With the aim of collaboratively elevating the voice of voluntary and community sector partners, Archipel OHT deliberately implemented a sustainable integrated governance strategy that encourages community and voluntary sector leadership and equitable shared decision-making. This perspective paper describes applied strategies, lessons learned, and efforts to maintain an environment that prioritizes their representation.

## Using Governance to Elevate Voluntary and Community Sector Representation

Like other healthcare systems, Canada is working to improve accessibility and overcome inefficient care delivery [1]. To achieve this, an integrated care approach is a potential remedy that uses coordinated, patient-centered, and holistic efforts to create seamless care across health and social services. In 2019, the Ontario provincial government asked health and social service providers to organize themselves into integrated care delivery systems called Ontario Health Teams (OHTs) [1]. Collaborating providers were instructed to create their own governance structures (i.e., guidelines for decision-making processes and systems of accountability and leadership). OHTs require significant intersectoral efforts, especially between acute care and the voluntary and community sector (VCS) [2,3]. Governance structures that support successful intersectoral collaboration are still being explored [2].

In 2023, Archipel OHT integrated 68 partners, including four hospitals and 34 VCS members (representing half the team), to optimize the delivery of health and social services to over 200,000 clients in the local regions of Ottawa East and Prescott-Russell. VCS partners offer a wide range of services including but not limited to social support networks, housing and food assistance, mental health support, and hospice and home care. Client partner representatives who have lived experience as services users also volunteer at Archipel OHT. This perspective paper describes governance structures and ongoing efforts to prioritize VCS representation. Insights can be applied to other integrated care systems aligned with this objective. Perspectives are from a team with lived experience at Archipel OHT, including community representatives in co-leadership roles (DV, SL), an Executive Director (ET), and a student working group collaborator (AB). We are also informed by discussions with past and current partners (see Acknowledgements) and grounded in evidence-based literature.

## Unequal Power Dynamics within Integrated Teams

Governance structures for integrated care teams should address unequal intersectoral and disciplinary power dynamics. Compared to the acute care sector, there are financial and operational restrictions in the VCS that contribute to unequal intersectoral power dynamics [3,4]. VCS partners often have less funding and human resources, especially those strictly from social or voluntary organizations [3,4]. Interdisciplinary power dynamics are also important to note. Integrated care teams need mid-level, community-based professionals who have expertise in holistic, patient-centered care [5,6]. These professionals are often from female-dominated disciplines [5,6]. In general, women are poorly represented in healthcare leadership [7] and physicians are historically perceived as higher in the healthcare hierarchy [7,8]. Both gendered healthcare leadership disparities and interdisciplinary tensions have been shown to affect collaboration [8].

## Sustainable Integrated Governance

VCS expertise and resources are necessary to effectively coordinate seamless, holistic care delivery. The health and well-being of a population extends far beyond care that requires a hospital. The VCS is equipped to meet on-going health and social service needs of communities. However, the unequal power dynamics (described previously) risk disrupting the VCS’s ability to contribute meaningfully on integrated care teams. In past collaborations between the current partners of Archipel OHT, feelings of unequal power dynamics and hospital dominance were expressed by VCS members. To counter this, Archipel OHT consciously committed to fostering VCS leadership from the outset. A sustainable integrated governance framework was designed to support trusted and inclusive collaboration. Here, sustainability refers to successful system integration. Examples of strategies explored and implemented include:

- Consensus decisions (rather than voting) to explore concerns and resource sharing/management
- Power-holding partners recognizing their privileged position
- Establishing one representative voice per sector (see below)
- Including the VCS through purposeful effort (i.e., outreach before decision-making)
- Prioritizing voices in the following order: client partner representatives, the VCS, and lastly acute care partners

## Strategies & Lessons Learned

### Structures to Support the VCS

Archipel OHT’s governance is structured around a steering committee (i.e., forum for strategic decisions, planning, and determining shared goals) with two co-leads and eleven collaborating domains. Each domain has one representative (e.g., four hospital partners hold one spot), aside from primary care which has two. Co-leads (DV, SL) are women and nurses in community-based leadership roles, giving direct power to the VCS to bring concerns forward while supporting women and *mid-level* professionals in authority holding positions.

### Putting in the Relational Work

Establishing intersectoral trust and mutual respect through relational work by VCS leaders was essential to increase partner engagement [2,3,9]. Relational trust is foundational to integrated care governance [3]. VCS partners needed reassurance that they were not token members. Hospital and Archipel leaders nurtured an intersectoral relationship that was not perceived as competitive. Clinical co-leads from the VCS acted as liaisons for members unfamiliar with hospital partners. Relationships established during previous hospital work meant co-leads personally trusted hospital partners, instilling confidence and bridging intersectoral gaps. Acute care partners sometimes did not realize their privileged position, unintentionally reinforcing unequal power dynamics by not adapting to VCS partner needs (e.g., slowing decision-making processes when VCS partners did not have capacity to participate). Relationships among OHT members, especially between co-leads and hospital executives, enabled honest conversations when these issues occurred.

### Making Involvement Accessible for the VCS

VCS partners can have a reduced capacity to participate on integrated care teams due to workload and resources constraints [3,4]. Therefore, additional efforts are made to facilitate their participation. Equitable shared decision-making means following a balanced and fair process where partners with less power are included through active and intentional support. For instance, decisions are strategically delayed until consensus is achieved, and outreaching is done to involve important but absent partners. Additionally, integrated care partners’ training and education is often specialized according to their role, meaning language, concepts, and acronyms used may be unfamiliar to other partners [3,10]. To support diverse partner engagement, shared language is established to keep partners on the same page conceptually, including explaining and limiting sector and/or discipline specific jargon [9,10]. Making participation accessible in these ways promotes meaningful engagement [10].

## Recommendations Based on Lessons Learned

Governance should be designed to support the VCS from the outset when possible. Structuring leadership roles around VCS representatives will help to ensure they are in a position of authority. If these leaders have multi-sectoral experience, this will also help to nurture intersectoral relationships and create trust. When unequal power dynamics exist, they must be addressed with open and honest conversations to bring awareness of privilege to dominant partners. Integrated teams must also accommodate the realities faced by the VCS workforce who often have a reduced capacity to participate – seek out their input directly and pause decisions as needed. Reduced capacity does not justify neglecting their input. Finally, establishing a shared language is helpful to ensure intersectoral and interdisciplinary partners are on the same page conceptually.

## Conclusion

To challenge unequal power dynamics, Archipel OHT follows a sustainable integrated governance framework. Core strategies include equitable shared decision-making, VCS leadership, establishing relational trust, and adapting to accommodate the VCS. Foregrounding the VCS requires on-going coaching and intentional effort from leadership. Integrated care governance with concern for power dynamics can help overcome historical tensions of disciplinary and intersectoral collaboration and support meaningful partner engagement. Ultimately, this will serve as a foundation for more balanced decisions that benefit underrepresented communities often served by the VCS, forwarding access to a better system built for and by system users.
